# Comparison of Fatigue Properties and Fatigue Crack Growth Rates of Various Implantable Metals

**DOI:** 10.3390/ma5122981

**Published:** 2012-12-19

**Authors:** Yoshimitsu Okazaki

**Affiliations:** Advanced Biomaterials Group, Human Technology Research Institute, National Institute of Advanced Industrial Science and Technology, 1-1-1 Higashi, Tsukuba-shi, Ibaraki 305-8566, Japan; E-mail: y-okazaki@aist.co.jp; Tel.: + 81-29-861-7179; Fax: + 81-29-861-6149

**Keywords:** implantable metal, mechanical property, fatigue property, notch effect, heat treatment, stress intensity factor, fatigue crack growth rate

## Abstract

The fatigue strength, effects of a notch on the fatigue strength, and fatigue crack growth rate of Ti-15Zr-4Nb-4Ta alloy were compared with those of other implantable metals. Zr, Nb, and Ta are important alloying elements for Ti alloys for attaining superior long-term corrosion resistance and biocompatibility. The highly biocompatible Ti-15Zr-4Nb-4Ta alloy exhibited an excellent balance between strength and ductility. Its notched tensile strength was much higher than that of a smooth specimen. The strength of 20% cold-worked commercially pure (C.P.) grade 4 Ti was close to that of Ti alloy. The tension-to-tension fatigue strength of an annealed Ti-15Zr-4Nb-4Ta rod at 10^7^ cycles was approximately 740 MPa. The fatigue strength of this alloy was much improved by aging treatment after solution treatment. The fatigue strengths of C.P. grade 4 Ti and stainless steel were markedly improved by 20% cold working. The fatigue strength of Co-Cr-Mo alloy was markedly increased by hot forging. The notch fatigue strengths of 20% cold-worked C.P. grade 4 Ti, and annealed and aged Ti-15Zr-4Nb-4Ta, and annealed Ti-6Al-4V alloys were less than those of the smooth specimens. The fatigue crack growth rate of Ti-15Zr-4Nb-4Ta was the same as that of Ti-6Al-4V. The fatigue crack growth rate in 0.9% NaCl was the same as that in air. Stainless steel and Co-Cr-Mo-Ni-Fe alloy had a larger stress-intensity factor range (ΔK) than Ti alloy.

## 1. Introduction

Orthopedic implants should exhibit biomechanical and biochemical compatibility as well as biological safety. Therefore, many types of metallic orthopedic devices, which are made of metallic materials with excellent mechanical properties and structural stability, are used worldwide in orthopedic [1]. Ti alloys are categorized into three types according to their microstructure: α (alpha)-type alloy having a hexagonal close-packed structure, hcp; β (beta)-type having a body-centered cubic structure, bcc; and α-β-type having a mixed structure of the α- and β-phases [1]. Among α-β-type Ti alloys, Ti-6Al-4V alloy has various orthopedic applications. α-β-type Ti alloys demonstrate better fatigue characteristics than β-type alloys [1]. Another α-β-type Ti alloy, Ti-15Zr-4Nb-4Ta alloy, has been developed in Japan as a highly biocompatible alloy for long-term biomedical applications [1,2,3]; it is now standardized in the Japanese Industrial Standards (JIS) T 7401-4 [4].

Fatigue is a type of damage often observed in implanted metals and is regarded as an important cause of orthopedic metal implant failure [5,6,7,8,9,10,11,12,13,14,15,16,17,18,19,20,21,22,23,24,25,26,27,28,29,30,31,32,33,34]. To summarize the literature on causes of failure, prosthetic fracture occurs owing to fatigue due to stress concentration that develops near holes of plates and rods, screws, spinal implants, and junctions of artificial hip stems. In particular, the existence of a notch markedly reduces the fatigue strength of materials. In developing highly durable devices, testing the fatigue strength of materials is crucial for predicting implant durability. A few studies have compared fatigue properties among implantable metals using specimens prepared by the same manufacturing process under the same experimental conditions. As fatigue testing methods, several standard methods have been established in JIS and the American Society for Testing and Materials (ASTM). JIS T 0309 [35] and ASTM F 1801 [36] provide methods for investigating the fatigue of metallic implant materials. JIS T 0310 [37] standardizes the fatigue testing method for the notch sensitivity and fatigue crack growth properties of metallic biomaterials. Our fatigue test was based on these standardized testing methods. The fatigue strength and effects of a notch on the fatigue strength and fatigue crack growth rate of Ti-15Zr-4Nb-4Ta alloy were compared with those of other implantable metals, namely, commercially pure (C.P.) grade 4 Ti, Ti-6Al-4V, Co-Cr-Mo, Co-Cr-Mo-Ni-Fe, and Co-Cr-W-Ni alloys, and stainless steels.

In this study, we conducted fatigue tests on smooth and notched specimens, and fatigue crack growth tests on Ti materials, Co-Cr-Mo, Co-Cr-Mo-Ni-Fe, and Co-Cr-W-Ni alloys, and stainless steels. The highly biocompatible Ti-15Zr-4Nb-4Ta alloy was used to determine the extent to which the fatigue strength and fatigue crack growth rate are affected by the specimen configuration (plate or rod), manufacturing process, heat treatment, and stress intensity factor. To evaluate the effects of forging, heat treatment, and stress intensity factor on smooth and notched fatigue strengths, the S-N curves (maximum stress *vs.* number of cycles) of Ti-15Zr-4Nb-4Ta alloy were compared with those of C.P. grade 4 Ti, Ti-6Al-4V, Co-Cr-Mo, two types of Co-Cr-Mo-Ni-Fe, and Co-Cr-W-Ni alloys, and three types of stainless steel. Tensile tests were also carried out on these materials at room temperature to examine the correlation between their mechanical and fatigue strengths. The effects of an R-notch and a V-notch on notched fatigue strength were examined. The fatigue crack growth rates of Ti-15Zr-4Nb-4Ta, Ti-6Al-4V, Co-Cr-Mo-Ni-Fe, and stainless steels were measured. Moreover, the maximum stress amplitude σ_th_ at which no crack propagation occurs, estimated from the threshold stress-intensity factor range (ΔK_th_) obtained by a fatigue crack growth test, was compared with the notched fatigue strength (σ_n_) at 10^7^ cycles.

## 2. Materials and Methods

### 2.1. Alloy Specimens and Heat Treatment

Vacuum-arc melting was performed on the Ti-15Zr-4Nb-4Ta alloy (JIS T 7401-4) [4]. A Ti-15Zr-4Nb-4Ta alloy ingot was homogenized at approximately 1200 °C for more than 6 h and β-forged at the same temperature to a forging ratio (cross section before forging/cross section after forging) of more than 3. Then, β-forging, while controlling the grain growth of the β-phase, was conducted at 1050 to 1100 °C to make the β-phase as small as possible, in proportion to the size of the billet and forging ratio. Afterwards, α-β-forging at T_β_-(35 to 50 °C) was conducted to obtain the α- and β-structures by decoupling the fine β-phase. T_β_ indicates the β-transus temperature (100 vol% β phase). 100 and 50 mm square billets were manufactured by α-β-forging [1]. Some of the Ti-15Zr-4Nb-4Ta billets were α-β-rolled into rods or plates. After α-β forging or α-β rolling, all the Ti billets, rods, and plates were annealed at 700 °C for 2 h. Some of the α-β-forged Ti-15Zr-4Nb-4Ta billets, rods, and plates were solution-treated at 785 ºC for 1 h and then quenched in water. After the solution treatment, these Ti-15Zr-4Nb-4Ta billets, rods, and plates were aged at 400ºC for 8 h and then cooled in air (aged Ti-15Zr-4Nb-4Ta).

To compare the fatigue properties of the alloys with those of other implantable metals, vacuum-arc melting was performed on α-type C.P. grade 4 Ti (ISO 5832-2) [38] and (α + β)-type Ti-6Al-4V alloy used for medical implants (ISO 5832-3) [39]. All the fabricated ingots were soaked and β-forged into billets and rods under the following conditions: 1100 °C-3 h for C.P. grade 4 Ti and 1150 °C-3 h for Ti-6Al-4V. The billets and rods were then α-β-forged (starting temperatures: 850 °C for C.P. grade 4 Ti and 930 °C for Ti-6Al-4V). Some of the C.P. grade 4 Ti billets were cold-rolled to prepare 20% reduced C.P. grade 4 Ti (20% cold-rolled C.P. grade 4 Ti). Moreover, three types of stainless steel were prepared by vacuum-induction melting and mechanical alloying: ISO 5832-1 stainless steel [40], 316 L stainless steel, and high-nitrogen (high-N) stainless steel specified in ISO 5832-9 [41]. Cold-rolled ISO 5832 billets were solution-treated at 1075 °C for 30 min and then quenched in water (solution-treated ISO 5832). Some of these solution-treated billets were cold-rolled to prepare 20% reduced ISO 5832 (20% cold-rolled ISO 5832). 316 L rod bars were solution-treated at 1050 °C for 2 min and then quenched in water (solution-treated 316 L). A high-nitrogen stainless steel was also prepared in a powdered state by mechanical alloying in a nitrogen gas atmosphere. The powder was hot-pressed into rods at 1050 °C. The rod specimens were solution-treated at 1150 °C for 1 h and then quenched in water (solution-treated high-N) [42]. For comparison, a Co-Cr-Mo (ISO 5832-4) ingot (As-cast) was hot-forged into rod specimens and then annealed at 1200 °C for 1 h (annealed). After holding them at 1150 °C for 1 h, some of these annealed rods were hot-forged to a 60% reduction in area (hot-forged). Moreover, the hot-forged rods were hot-forged to a 60% reduction in area after holding them at 1000 °C for 1 h (warm-worked) [43]. High-carbon (high-C, 0.12%C) and low-carbon (low-C, 0.06% C) Co-Cr-Mo-Ni-Fe alloys, which are specified in ISO 5832-8, were also prepared by vacuum-induction melting. The hot-forged high-C rods were solution-annealed at 1200 °C for 15 min and then quenched in water. The solution-annealed high-C rods were cold-drawn to a 50% reduction in area (cold-drawn high-C). Some of the cold-drawn high-C rods were solution-annealed at 1130 °C for 5 min and then quenched in water (annealed high-C). The hot-forged low-C rods were solution-annealed at 1000 °C for 0.5 h and then quenched in water. They were then additionally cold-drawn to a 50% reduction in area (cold-drawn low-C) [44]. Moreover, a Co-Cr-W-Ni (ASTM F90) ingot was hot-forged into rod specimens [45]. The forged rods were solution-treated at 1075 °C for 1 h and then quenched in water. Table 1 shows the chemical compositions of the materials used in this study.

**Table 1:** Chemical compositions (mass%) of materials used.

Alloy	Chemical compositions (mass%)													
	Zr		Nb		Ta	Pd	Al	V	Fe	O	N	H	C	Ti
Ti-15Zr-4Nb-4Ta	15.52		4.0		4.0	0.18	-	-	0.026	0.20	0.042	0.0011	<0.005	Bal.
C.P. grade 4 Ti	-		-		-	-	-	-	0.197	0.275	0.003	0.0069	0.011	Bal.
Ti-6Al-4V	-		-		-	-	6.40	4.40	0.10	0.07	0.02	0.0027	0.025	Bal.
**Alloy**	**Cr**	**Nb**	**Mo**	**Ni**	**Cu**	**Mn**	**Fe**	**P**	**N**	**W**	**C**	**S**	**Co**	**Si**
Stainless Steel														
316L	17.0	-	2.04	12.15	-	1.40	-	0.034	-	-	0.024	0.009	-	0.82
ISO (5832-1)	18.33	-	3.48	14.56	<0.01	1.77	Bal.	0.016	0.157	-	0.021	0.0014	-	0.79
High-N Stainless	21.63	0.54	2.43	9.72	0.08	2.88	Bal.	0.013	0.311	-	0.022	0.007	-	0.75
Co-Cr-Mo	27.82	-	6.23	0.51	-	0.6	0.68	-	-	-	0.26	-	Bal.	0.6
Co-Cr-Mo-Ni-Fe														
High C	20.77	-	7.88	14.51	-	1.27	Bal.	0.005	-	-	0.12	0.0025	39.7	0.85
Low C	19.33	-	6.74	16.65	-	1.78	Bal.	<0.005	-	-	0.06	0.0006	40.1	0.47
Co-Cr-Ni-W	20.49	-	0.23	10.52	0.01	1.32	1.69	0.003	-	14.97	0.09	0.002	Bal.	0.14

### 2.2. Tensile Test

To establish baseline data for fatigue testing, smooth and notched mechanical tests were performed on each alloy. Three smooth tensile samples were prepared in accordance with JIS standard test methods for the tension testing of metallic materials (JIS H 4600) [46]. For each alloy, specimens of 8 mm diameter and 40 mm gauge length were pulled at a crosshead speed of 1 mm/min until a 0.2% proof strength was reached. The crosshead speed was then changed and maintained at 2.5 mm/min until the specimen fractured. Ultimate tensile strength (σ_UTS_), 0.2% proof strength (σ_0.2%PS_), the percentage of total elongation (%T.E.) to fracture, and the reduction in area (%R.A.) were determined.

In the ASTM test method for the sharp V-notch tension testing of cylindrical specimens (E 602) [47], since the onset of plastic deformation occurs at the proof strength, it is widely recognized that the ratio of the notch tensile strength to the smooth 0.2% proof strength is a more useful predictor of toughness. V-notched tensile samples of annealed Ti-15Zr-4Nb-4Ta alloy were prepared with a stress intensity factor (K_t_) of 3.2 (Figure 2c) and a notch root diameter of 6 mm. Notch tensile testing was performed according to ASTM E 602. Notch tensile strength (σ_NTS_), the ratio of σ_NTS_ to σ_UTS_, and the ratio of σ_NTS_ to σ_0.2%PS_, as required by ASTM E602, were determined.

### 2.3. Specimens for Fatigue Test

**Figure 1:**
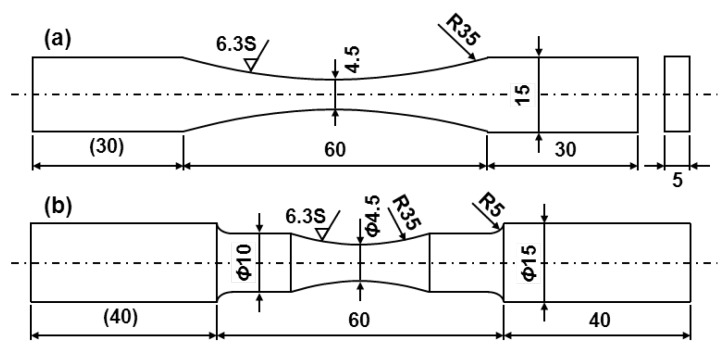
Dimensions of smooth fatigue test specimens. (**a**) Plate; and (**b**) Rod specimens with continuous radius between ends (hourglass-shaped plate and rod).

**Figure 2:**
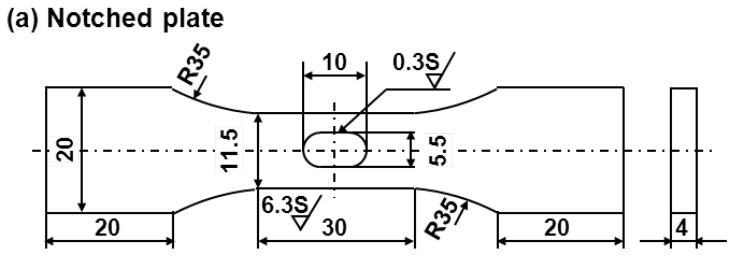

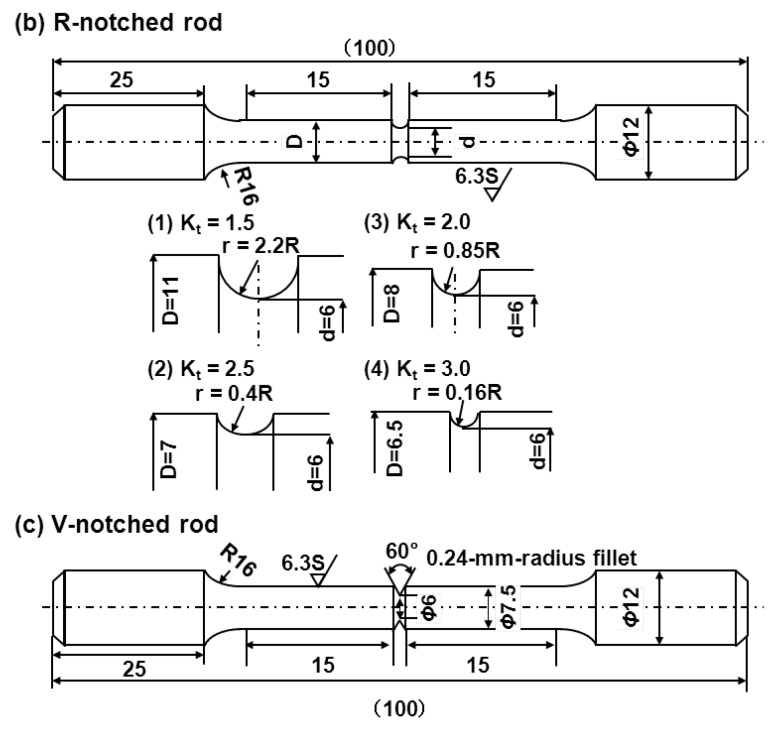
Dimensions of notch fatigue test specimens. (**a**) Notch plate specimen (K_t_ = 1.8); (**b**) R-notched (K_t_ = 1.5, 2.0, 2.5, and 3.0); and (**c**) V-notched (K_t_ = 3.2) rod specimens.

Smooth fatigue test specimens were prepared in accordance with JIS T 0309. Figure 1 shows two types of specimen cut from annealed and aged Ti-15Zr-4Nb-4Ta alloys and various other implantable metals. Such specimens with rectangular and circular cross sections (plates and rods, respectively) were used as the test specimens; they have a continuous radius between their ends (hourglass-shaped plates and rod specimens). The specimens were machined with their longitudinal direction parallel to the forging direction. To remove the inner strain formed on the surfaces during manufacture, the surfaces were fully ground in the direction parallel to the test specimen using 600 and 1200 grit waterproof emery papers. Notch fatigue test specimens were prepared in accordance with JIS T 0310. R- and V-notched tensile samples with stress intensity factors (K_t_) of 1.5, 1.8, 2.0, 2.5, 3.0, and 3.2 were prepared as shown in Figure 2. The stress intensity factor of the specimens was calculated in accordance with the relationship between stress intensity factor and notch configuration [*i.e.*, the diagram showing the relationship between K_t_ and the ratios of r/D and d/D (r: radius fillet, d: notch root diameter, and D: specimen diameter)] in the stress concentration design factor handbook [48].

### 2.4. Fatigue Test

Smooth and notch fatigue tests were conducted in accordance with JIS T 0309 and JIS T 0310, respectively, using an electrohydraulic servo testing machine in air or Eagle’s medium at 37 °C. For the test in the solution, the specimen was fitted into a polyethylene testing cell containing Eagle’s medium and then set on a fatigue testing machine. The solution temperature inside the cell was maintained at 37 °C using heated water circulating around the cell. The tests were carried out in the tension-to-tension mode with a sine wave. The stress ratio [R = (minimum stress)/(maximum stress)] was 0.1 and the wave frequency was 10 Hz. S-N curves [maximum stress (maximum applied load/area of cross section) *vs.* number of cycles] of the various specimens were measured. The fatigue notch factor (K_f_) and fatigue notch sensitivity (q) were estimated from the S-N curves of each alloy. K_f_ is the ratio of the fatigue strength of a smooth sample to the notched fatigue strength at 10^7^ cycles [*i.e.*, K_f_ = (σ_Smooth_)/(σ_Notch_)]. The fatigue notch sensitivity (q) was calculated using q = ( K_f_ − 1)/(K_t_ − 1) [49].

### 2.5. Fatigue-Crack Growth Test

**Figure 3:**
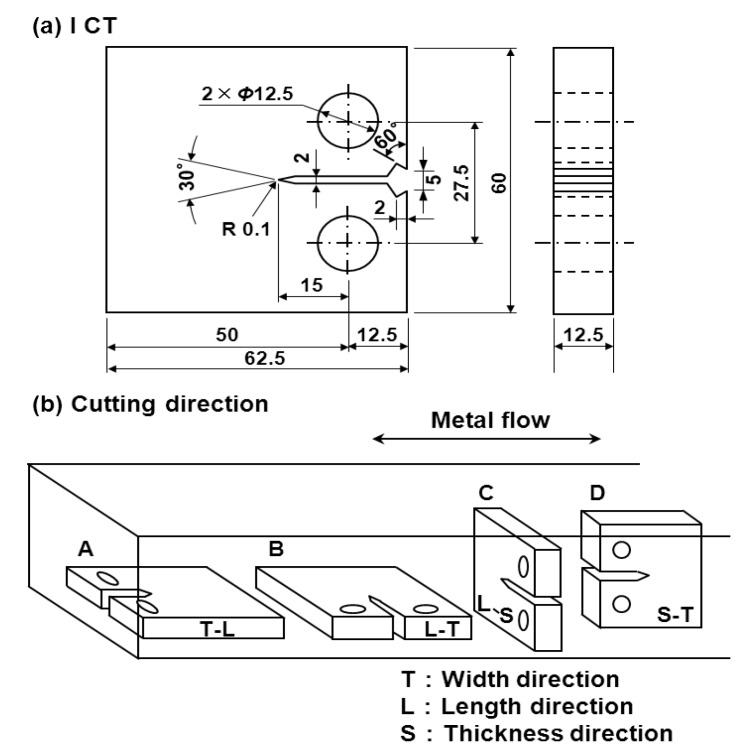
(**a**) Dimensions of compact test (CT) specimens; and (**b**) cutting directions.

The fatigue crack growth test was conducted in accordance with JIS T 0310. To investigate the effect of heat treatment (microstructure) on fatigue crack growth properties, the following four treatments were performed on Ti-15Zr-4Nb-4Ta billets: β-annealing at 1100 °C for 2 h and then air-cooling (β-annealed, acicular structure); α-β forging at 800 °C after β-annealing (α-β-forged structure); annealing at 650 °C for 2 h after α-β forging and then air-cooling (annealed α-β granular structure); solution treatment at 785 °C for 1 h, quenching in water, aging at 400 °C for 8 h, and then cooling in air (aged structure). The compact tension (CT) specimen shown in Figure 3a was cut from four types of heat-treated Ti-15Zr-4Nb-4Ta (β-annealed, α-β forged, annealed, and aged), annealed Ti-6Al-4V, annealed Co-Cr-Mo-Ni-Fe alloys, and solution-treated ISO 5832 stainless steel. Moreover, to investigate the effect of the cutting direction on fatigue crack growth properties, CT specimens were cut from four directions (L-T, T-L, S-T, and S-L) as shown in Figure 3b.

**Figure 4:**
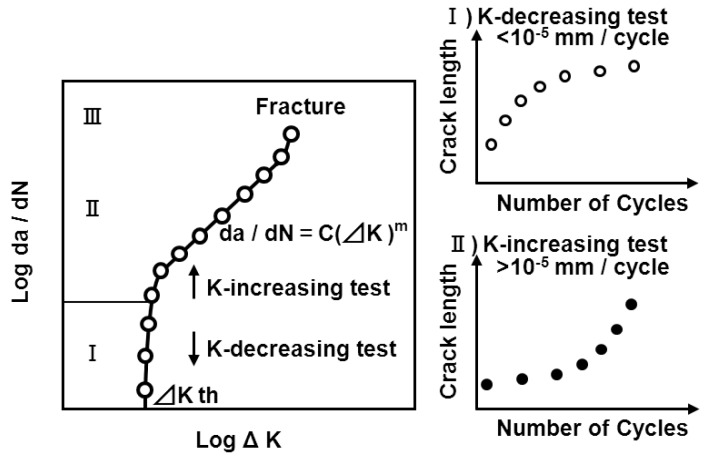
Testing method for fatigue crack growth properties.

Fatigue crack growth properties are shown in Figure 4. The longitudinal axis represents the fatigue crack growth rate (da/dN), and the abscissa the stress-intensity factor range (ΔK). Figure 4 is divided into three regions. The fatigue crack growth rate is very low in Region I, and the crack rate is deemed not to increase practically below a certain ΔK. This ΔK is referred to as the threshold stress-intensity factor range and is indicated as ΔK_th_. ΔK_th_ is generally represented as ΔK at da/Dn = 10^7^ mm/cycle [37,50]. Region II is the range where the crack grows stably, and a linear relationship, referred to as the Paris expression, exists between da/dN and ΔK when they are expressed as a double logarithm; the formula is represented as da/dN = C(ΔK)^m^, where C and m are material constants and ΔK = K_max_ − K_min_, which is the difference between the maximum and minimum stress intensities in a fatigue cycle, *i.e.*, the stress range. Stress intensity can be expressed by $\text{K}\;=\;\sigma \sqrt{\pi \alpha }\text{F}(\text{a}/\text{W})$, where *σ* represents the applied stress, and F is a dimensionless function of crack length (a) and the length of the cracked body (W) and depends on the specimen/object geometry [37,50]. For the compact-tension specimens used in this study, the stress-intensity range becomes:

ΔK = K_max_ − K_min_ = (1 − R)K_max_ (1)

R = Kmax/Kmin = Pmax/Pmin
(2)

ΔP = P_max_ − P_min_ = (1 − R) P_max_ (3)

(4) $$\Delta \text{K}=\Delta \text{P}(2+\alpha )(0.866+4.64\alpha -13.32\alpha^{2}+14.72\alpha^{3}-5.6\alpha^{4})/\text{B}/\sqrt{\text{w}}/{\left(1-\alpha \right)}^{3/2}$$

where ΔP is the load range, B is the sample thickness, and α = a/W. Fatigue crack growth in region II is measured using the constant-force amplitude test, which applies a constant force on the specimen repeatedly. However, that in region I cannot be measured using the constant-force amplitude test for the following reasons. When a fatigue crack growth test is conducted, it is necessary to create a precrack on the tip of the machined notch. However, when force for the actual fatigue crack growth test is reduced to more than its acceptable level, the fatigue crack stops propagating owing to compressive residual stress formed on the crack tip. In addition, fatigue precrack creation at a smaller ΔK takes a very long time until a fatigue crack is induced. Thus, JIS T 0310 and ASTM E647 state the calculation procedure for the fatigue crack growth rate obtained by the K-decreasing test. The K-decreasing test is, as shown in Figure 4, a method of calculating the fatigue crack growth rate in region I by decreasing K exponentially with crack propagation, while slightly shedding off the load up to the extent that the prior loading history does not affect the fatigue crack growth property. There are differences in the test method used between regions I and II. When calculating fatigue crack growth properties in regions I and II with one CT specimen with an inner area of 60 × 62.5 mm^2^, crack growth in region I was first measured, and then, after increasing the load, crack growth in region II was measured. In region III, crack growth is rapid owing to cleavage or unstable-ductile fracture. In this work, the fatigue crack growth rate was only measured in regions I and II.

**Figure 5:**
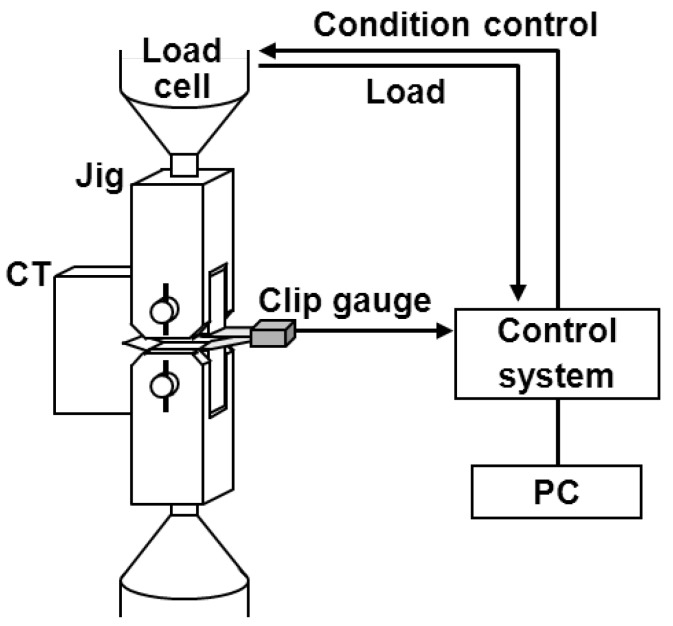
Fatigue crack growth test jig and equipment used.

**Figure 6:**
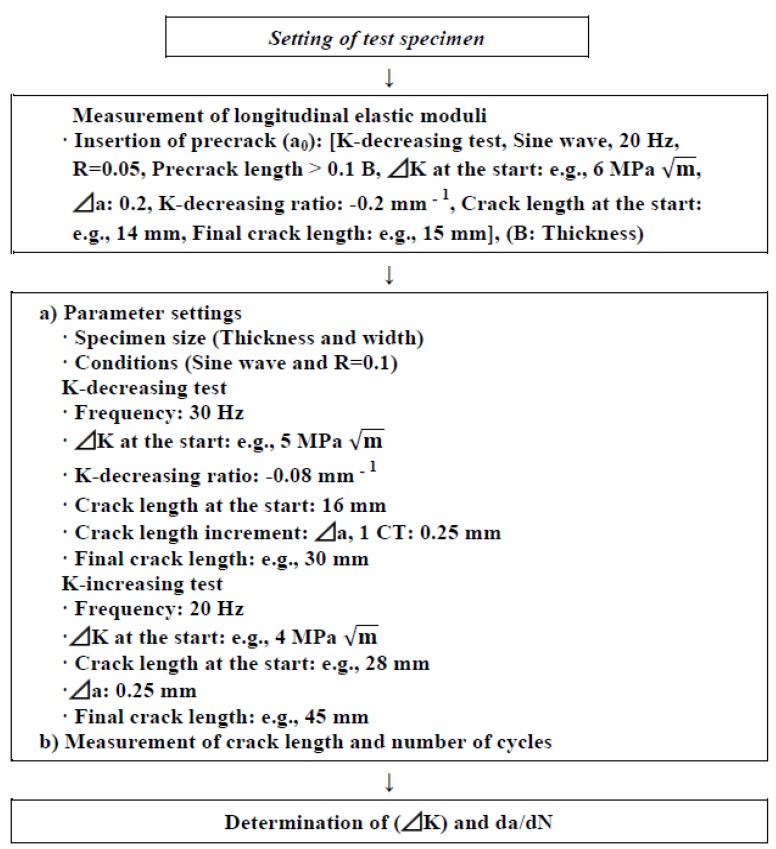
Fatigue crack growth test procedure.

Figure 5 shows the fatigue crack growth test jig in air atmosphere and the equipment used in this study. For the test in the solution at 37 °C, the specimen was placed in a testing cell containing 0.9% NaCl and then set on the transverse-mounted testing machine. The solution at 37 °C was circulated into the cell. A conceptual diagram of the method is shown in Figure 6. The fatigue crack length (a) was determined by the compliance method using a computer. Compliance is the ratio of the crack opening displacement δ to the load P (δ/P); the longer the crack, the larger the compliance. The relationship between compliance and crack length is formulated, and the crack length is calculated automatically from the compliance. A constant-force K-decreasing test was conducted under the conditions shown in Figure 6 in accordance with JIS T 0310 and ASTM E647. First, a precrack was introduced by the K-decreasing method, reducing the force automatically with crack propagation; then, the fatigue crack growth rate of da/dN = 10^−5^ − 10^−2^ mm/cycle was calculated by the K-increasing method, increasing K with crack propagation. When calculating a full curve for one specimen, the K-decreasing method, while reducing the force automatically with crack propagation, was applied to calculate the crack growth rate in region I down to ΔK_th_. The force ratio (R = minimum force/maximum force) was 0.1. The frequency in the fatigue precrack introduction test was 20 Hz, and that in the actual fatigue crack growth test was 30 Hz. The fatigue crack growth rate was calculated as a tangential line of the curve indicating the relationship between crack length (a) and cycle number (N) (da/dN). The fatigue crack growth system using a PC (Figure 5) was used for smoothing the A and N relation data by the seven-point approximation method in accordance with ASTM E647. The seven-point approximation method determines the second-order polynomial factor by the least-squares method in the range of a_i-3_ ≦ a ≦ a_i + 3_ after adapting seven each of the a and N successive digital data points to a second-order polynomial factor.

a_i_ = b_0_ + b_i _(N_i − _c_1_)/c_2_ + b_2_(N_i − _c_1_)^2^/c_2_ (5)

where b_0_, b_i_, and b_2_ are factors;

c_1_ = (N_−3_ + N_i + 3_)/2, and c_2_ = (N_i + 3_ − N_i + 3_)/2 (6)

Fatigue crack growth rate was calculated using a derivative of the above formula. Displacing each of the data points and grouping them into seven points were alternately performed to determine the fatigue crack growth rate.

## 3. Results and Discussion

### 3.1. Mechanical Properties of Various Implantable Metals

Table 2 shows a comparison of the mechanical properties of the various heat-treated implantable metals (highly biocompatible Ti-15Zr-4Nb-4Ta alloy, Ti-6Al-4V alloy, C.P. grade 4 Ti, three types of stainless steel, Co-Cr-Mo alloy, two types of Co-Cr-Mo-Ni-Fe alloy, and Co-Cr-Ni-W alloy). The means of 0.2% proof strength (σ_0.2%PS_), ultimate tensile strength (σ_UTS_), total elongation (T.E.), and reduction in area (R.A.), and their corresponding standard deviations were calculated for three test specimens of each material. The mechanical strengths of the implantable metals were markedly increased by a combination of manufacturing processes and heat treatment. In particular, the strength of the 20% cold-worked C.P. grade 4 Ti was close to that of the Ti alloy. The highly biocompatible Ti-15Zr-4Nb-4Ta alloy exhibited an excellent balance between strength and ductility. The mechanical strengths of the 20% cold-worked stainless steel, hot-forged and warm-worked Co-Cr-Mo, cold-drawn Co-Cr-Mo-Ni-Fe, and aged Ti-15Zr-4Nb-4Ta alloys were markedly higher than those of the solution-treated and annealed materials. On the other hand, the T.E. and R.A. values of the same materials decreased with an increase in their strengths. The values of σ_0.2%PS_, σ_UTS_, T.E., and R.A. from each of the Co-Cr-Mo alloy, CP-Ti grade 4, and Ti-6Al-4V alloy specimens were close to those reported in the literature [50,51,52]. Table 3 shows the notched tensile properties of the Ti-15Zr-4Nb-4Ta alloy. The results for the other materials except Ti-15Zr-4Nb-4Ta shown in Table 3 were cited from the literature [49]. The notched tensile strength (σ_NTS_) of the Ti-15Zr-4Nb-4Ta alloy was much higher than that of the smooth specimen. The σ_NTS_/σ_UTS_ and σ_NTS_/σ_0.2%PS_ values of each alloy were within the 1.4–1.5 and 1.6–1.8 ranges, respectively. The effect of the V-notch on the tensile strength of Ti-15Zr-4Nb-4Ta alloy at K_t_ = 3.2 coincided with the result reported in the literature [49].

**Table 2:** Mechanical properties of implantable metals and comparison of ratio of fatigue strength at 10^7^ cycles (σ_FS_) to σ_UTS_ among alloys.

Alloy	σ_0.2%PS_ (MPa)	σ_UTS_ (MPa)	T.E (%)	R.A (%)	σ_FS_/σ_UTS_
Ti-15Zr-4Nb-4Ta					
Annealed (Plate)	800 ± 14	910 ± 10	19 ± 2	-	0.54
Annealed (Rod)	848 ± 2	915 ± 3	21 ± 2	55 ± 3	0.80
Aged (Rod)	894 ± 5	1020 ± 8	15 ± 2	48 ± 3	0.89
C.P. grade 4 Ti					
Annealed	616 ± 6	700 ± 8	31 ± 4	44 ± 2	0.69
20% Cold-worked	859 ± 2	870 ± 2	14 ± 1	39 ± 3	0.50
Ti-6Al-4V	849 ± 1	934 ± 1	16 ± 1	42 ± 3	0.73
Stainless steel					
ISO 5832					
Solution-treated	338 ± 30	680 ± 8	68 ± 4	70 ± 2	0.55
20% Cold-worked	760 ± 5	878 ± 19	30 ± 11	70 ± 4	0.79
SUS 316L					
Solution-treated	262 ± 6	567 ± 2	56 ± 6	74 ± 7	0.67
High-N					
Solution-treated	436 ± 4	830 ± 4	37 ± 2	48 ± 7	0.57
Co-Cr-Mo					
As-cast	380 ± 30	550 ± 50	6 ± 3	14 ± 2	0.72
Annealed	552 ± 4	915 ± 15	20 ± 2	14 ± 1	0.75
Hot-forged	669 ± 40	1075 ± 20	7 ± 2	10 ± 2	0.74
Warm-worked	760 ± 5	1153 ± 16	12 ± 2	8 ± 2	0.84
Co-Cr-Mo-Ni-Fe					
High C					
Annealed	474 ± 4	1003 ± 4	65 ± 2	47 ± 2	0.64
Cold-drawn	1220 ± 56	1622 ± 22	12 ± 2	44 ± 4	0.68
Low C					
Annealed	410 ± 10	900 ± 4	38 ± 4	54 ± 4	-
Cold-drawn	1174 ± 45	1549 ± 22	9 ± 2	36 ± 5	0.69
Co-Cr-Ni-W					
Annealed	568 ± 30	1096 ± 34	47 ± 2	40 ± 2	0.62

**Table 3:** Comparison of smooth and notched tensile (K_t_ = 3.2) strengths obtained in this work and reported in the literature among different materials [49].

Material	Smooth tensile test		Notch tensile	Ratio	
	σ_0.2%PS_	σ_UTS_	σ_NTS_	σ_NTS_/σ_0.2%PS_	σ_NTS_/σ_UTS_
Ti-15Zr-4Nb-4Ta (Annealed rod)	848 ± 2	915 ± 3	1358 ± 17	1.60	1.48
SUS 316L	791 ± 11	1008 ± 7	1449 ± 3	1.83	1.44
C.P. grade 4 Ti	725 ± 8	888 ± 8	1310 ± 8	1.81	1.47
Ti-6Al-4V	827 ± 6	1024 ± 5	1426 ± 9	1.73	1.39

### 3.2. Fatigue Properties of Various Implantable Metals

**Figure 7:**
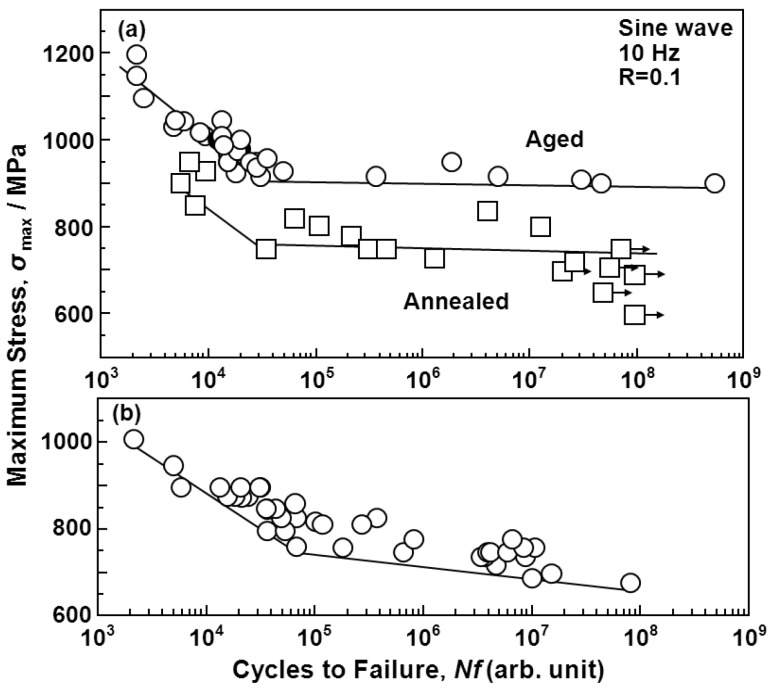
S-N curves obtained by tension-to-tension fatigue test in air for (**a**) annealed and aged Ti-15Zr-4Nb-4Ta alloys; and (**b**) annealed Ti-6Al-4V alloy rods.

**Figure 8:**
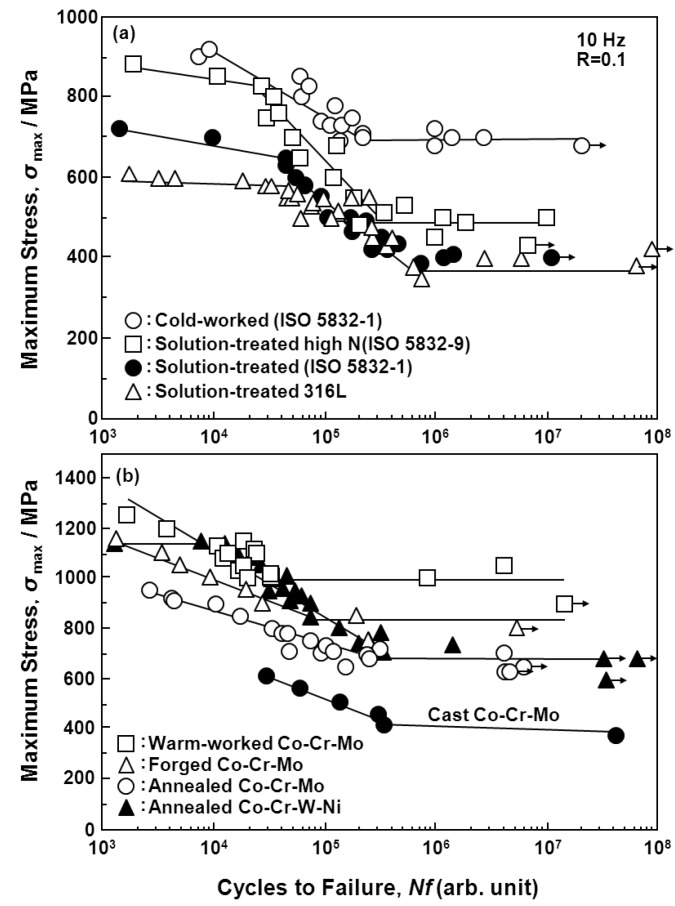
Comparison of S-N curves obtained by tension-to-tension fatigue test in Eagle’s medium at 37 °C for (**a**) stainless steels; and (**b**) Co-Cr-Mo alloys treated under various conditions.

**Figure 9:**
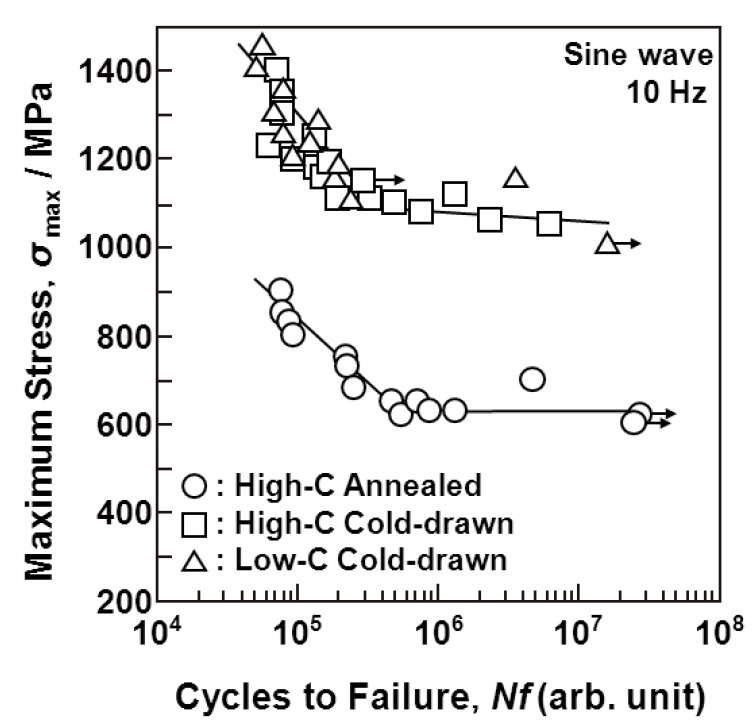
S-N curves obtained by tension-to-tension fatigue test with sine wave (10 Hz) for annealed high-C Co-Cr-Mo-Ni and 50% cold-drawn high-C and 50% cold-drawn low-C Co-Cr-Mo-Ni alloy rods.

Figure 7, Figure 8 and Figure 9 show the S-N curves obtained from the smooth hourglass-shaped rod specimens of the various treated implantable metals (annealed and aged Ti-15Zr-4Nb-4Ta alloys, annealed Ti-6Al-4V, solution-treated, and 20% cold-worked stainless steels, annealed, hot-forged, and warm-worked Co-Cr-Mo alloys, and annealed and cold-drawn Co-Cr-Mo-Ni-Fe alloys). Arrows in the figures indicate the lack of specimen fracture. For the Ti alloys, the fatigue strength (740 MPa) of Ti-15Zr-4Nb-4Ta at 10^7 ^cycles was slightly higher than that (680 MPa) of Ti-6Al-4V, as shown in Figure 7. The fatigue strength (approximately 700 MPa) of the stainless steel at 10^7 ^cycles was markedly improved by 20% cold working; it became close to that of the Ti alloy. The fatigue strength (approximately 1000 or 700 MPa) of the Co-Cr-Mo alloy at 10^7 ^cycles was markedly increased by warm working or hot forging; it was considerably higher than those of the Ti alloys (Figure 8). The fatigue strengths of the high-C and low-C Co-Cr-Mo-Ni-Fe alloys at 10^7 ^cycles were markedly improved by cold drawing compared with those of the annealed alloys; they were approximately 1100 MPa (Figure 9). The fatigue strength of the annealed Ti-15Zr-4Nb-4Ta alloy at 10^7^ cycles was higher than that of Ti-6Al-4V and β-type Ti-12Mo-6Zr-2Fe alloys in the literature (both strengths are approximately 600 MPa) [52]. Also, the fatigue strength of the aged Ti-15Zr-4Nb-4Ta alloy at 10^7^ cycles was higher than that of aged β-type Ti-29Nb-13Ta-4.6Zr alloy [53]. The ratios of the fatigue strength (σ_FS_) of these materials at 10^7 ^cycles to the ultimate tensile strength are shown in Table 2. The σ_FS_/σ_UTS_ values of all the materials were within the 0.5 to 0.9 range. The annealed and aged Ti-15Zr-4Nb-4Ta alloy rods exhibited particularly high σ_FS_/σ_UTS_ values of approximately 0.8 and 0.89, respectively.

### 3.3. Effect of Notch on Fatigue Strength of Various Implantable Metals

**Figure 10:**
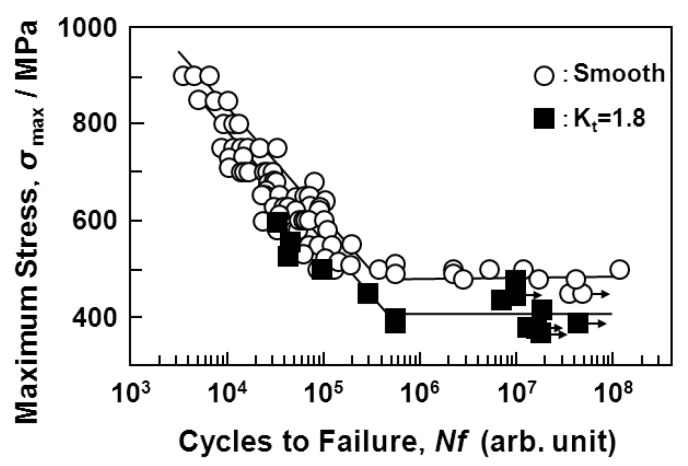
Effect of notch on S-N curves obtained by tension-to-tension fatigue test with sine wave (10 Hz) for annealed Ti-15Zr-4Nb-4Ta alloy plates.

**Figure 11:**
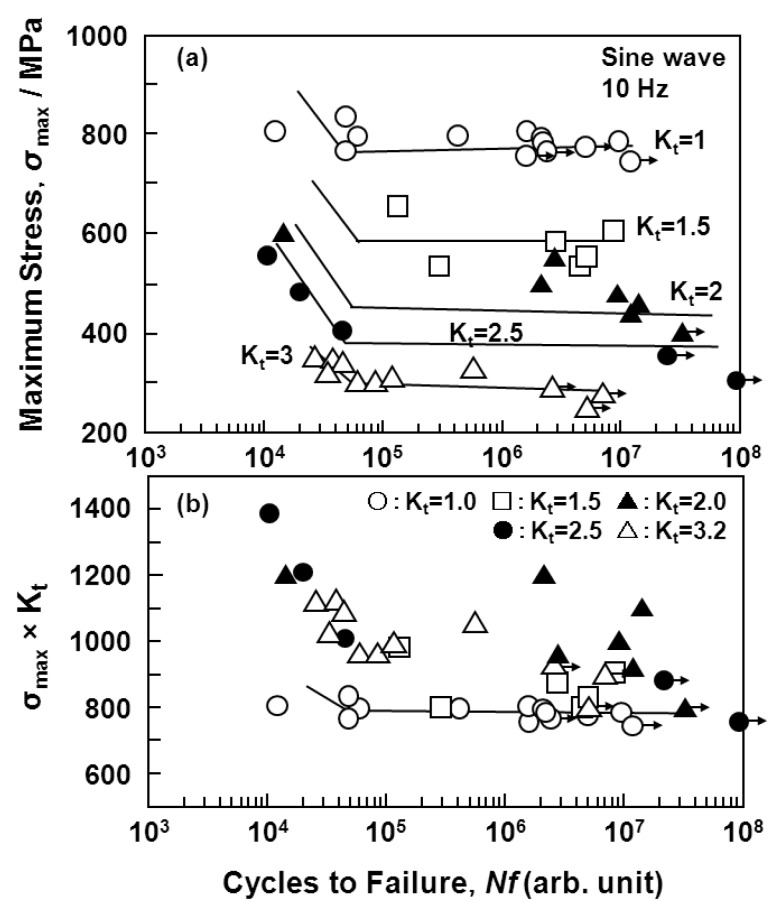
Effect of R-notch on S-N curves obtained by tension-to-tension fatigue test with annealed Ti-15Zr-4Nb-4Ta alloy rods. Effects of (**a**) stress intensity factor (K_t_); and (**b**) σ_max_ × K_t_ on S-N curves.

Figure 10 shows the effect of a notch (K_t_ = 1.8) on the S-N curves obtained from the annealed Ti-15Zr-4Nb-4Ta alloy plate. The fatigue strength of the alloy plate at 10^7 ^cycles slightly decreased with the existence of a notch. The effect of the stress intensity factor (K_t_ = 1.5, 2, 2.5, and 3) introduced by the R-notch on the S-N curves obtained from the annealed Ti-15Zr-4Nb-4Ta alloy rods is shown in Figure 11a. The fatigue strength at 10^7 ^cycles tended to decrease with increasing K_t_ from 1.5 up to 3. The effect of σ_max_ × K_t_ on the S-N curves of the same rods is shown in Figure 11b. In particular, at K_t_ less than 2, σ_max_ × K_t_ in the high-cycle region (*i.e.*, 10^7 ^cycles) of the S-N curves was close to the value of σ_max _(σ_FS_) obtained with the smooth specimens (K_t_ = 1). It is considered that these findings are useful for implant design, *i.e.*, for evaluating the durability of the stress concentration region of implant devices using σ_FS _(σ_max_) measured in smooth specimens and the stress intensity factor K_t_ in the stress concentration region.

**Figure 12:**
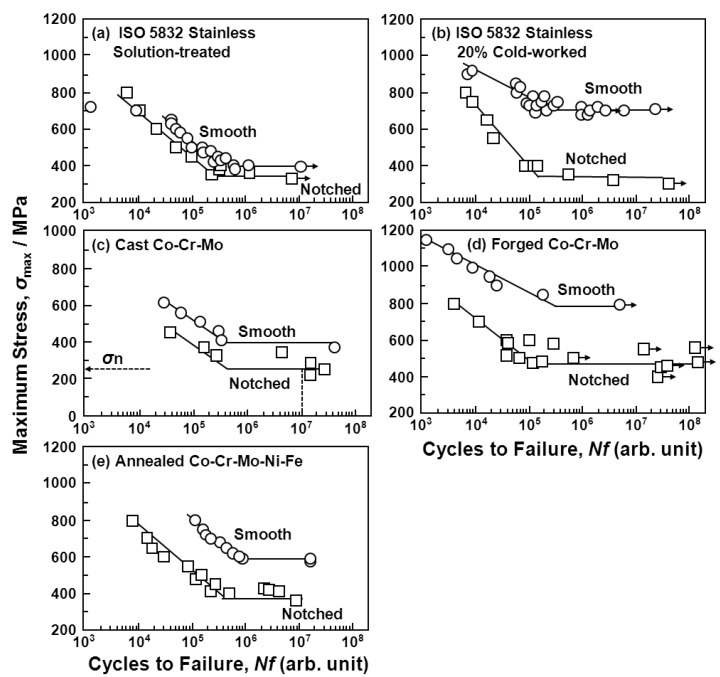
Effect of V-notch on S-N curves obtained by tension-to-tension fatigue test (sine wave, 10 Hz) for implantable metals.

**Figure 13:**
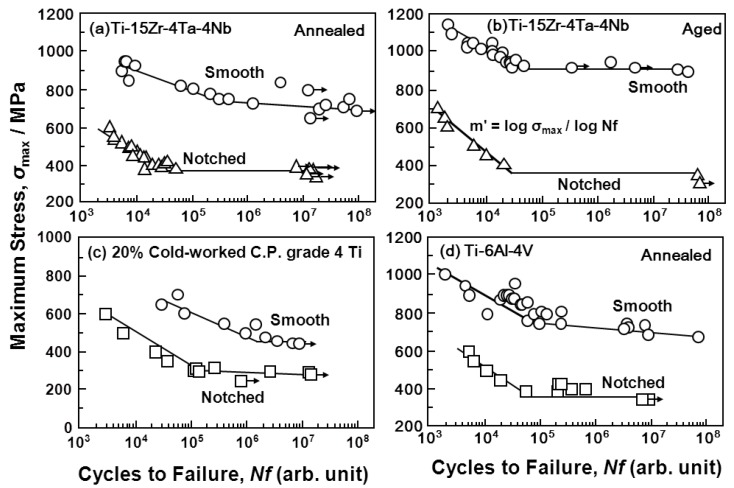
Effect of V-notch on S-N curves obtained by tension-to-tension fatigue test (sine wave, 10 Hz) for Ti alloys.

**Figure 14:**
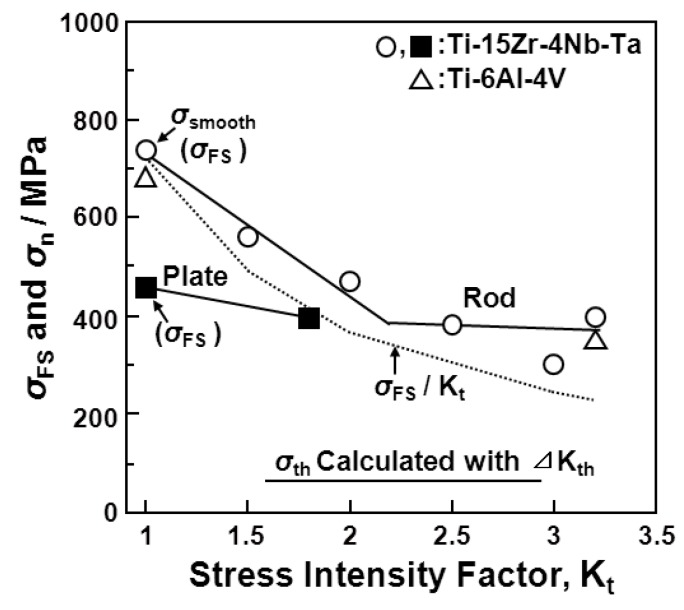
Effect of stress intensity factor (K_t_) on notched fatigue strength (σ_n_) and smooth fatigue strength (σ_FS_) at 10^7^ cycles obtained by tension-to-tension fatigue tests with annealed Ti-15Zr-4Nb-4Ta rods and plates and Ti-6Al-4V rods.

Figure 12 and Figure 13 show the effect of the V-notch (K_t_ = 3.2) on the S-N curves of the annealed Ti-15Zr-4Nb-4Ta alloy rods. The effect of the V-notch on the S-N curves of the solution-treated stainless steel (ISO 5832) was small. However, the fatigue strength of the 20% cold-worked stainless steel markedly decreased with the existence of the V-notch. The effect of the V-notch on the S-N curves of the cast and forged Co-Cr-Mo and Co-Cr-Mo-Ni-Fe alloys was observed, as shown in Figure 12c–e, respectively. The fatigue strengths of the 20% cold-worked C.P. grade 4 Ti, annealed and aged Ti-15Zr-4Nb-4Ta alloys, and annealed Ti-6Al-4V alloy at 10^7 ^cycles were markedly lower than those of the smooth specimens. Figure 14 shows the effect of K_t_ on the notched fatigue strength σ_n_ at 10^7 ^cycles obtained with the annealed Ti-15Zr-4Nb-4Ta and Ti-6Al-4V alloys. The σ_n_ of the V-notched Ti-15Zr-4Nb-4Ta rod decreased with increasing in K_t_ at K_t_ < 2. However, it showed no decrease above approximately K_t_ = 2. Also, the difference between σ_n_ and σ_FS_/K_t_ increased at 2 < K_t_ This might be due to a nonpropagating crack at the crack tip. The fatigue strength (500 MPa) of the annealed Ti-15Zr-4Nb-4Ta alloy with a notch (K_t_ = 2.0) at 10^7^ cycles was higher than that of Ti-6Al-4V and Ti-12Mo-6Zr-2Fe alloys with a notch (K_t_ = 1.6) reported in the literature (approximately 200 and 400 MPa, respectively) [52]. The fatigue notch factors (K_f_) and fatigue notch sensitivities (q) obtained in the notch fatigue test are summarized in Table 4. The q values of the R-notched Ti alloy were higher than those of the V-notched Ti alloy. The q value of the Ti-6Al-4V alloy obtained in this study was close to that of Ti-6Al-4V alloy reported in the literature (approximately 0.5) [53].

**Table 4:** Comparison of fatigue notch factor (K_f_) and fatigue notch sensitivity (q) at 10^7^ cycles among alloys.

Alloy		K_f_	q
Ti-15Zr-4Nb-4Ta			
Annealed plate (K_t_ = 1.8)		1.15	0.2
Annealed rod		-	-
R-notch	K_t_ = 1.5	1.42	0.84
	K_t_ = 2.0	1.80	0.80
	K_t_ = 2.5	2.30	0.86
	K_t_ = 3.0	2.6	0.8
Ti-15Zr-4Nb-4Ta			
V-notch	K_t_ = 3.2		
	Annealed rod	1.8	0.4
	Aged rod	2.5	0.7
Ti-6Al-4V			
V-notch	K_t_ = 3.2	2	0.45
	Annealed rod		
C.P. grade 4Ti			
K_t_ = 3.2 (20% Cold-worked)		1.55	0.25
ISO 5832 Stainless steel			
Solution-treated (K_t_ = 3.2)		1.29	0.13
20% Cold-worked (K_t_ = 3.2)		1.84	0.38
Co-Cr-Mo			
Cast (K_t_ = 3.2)		1.65	0.30
Forged (K_t_ = 3.2)		1.56	0.25
Co-Cr-Mo-Ni-Fe			
Annealed (K_t_ = 3.2)		1.59	0.27

### 3.4. Fatigue-Crack Growth Properties of Various Implantable Metals

**Figure 15:**
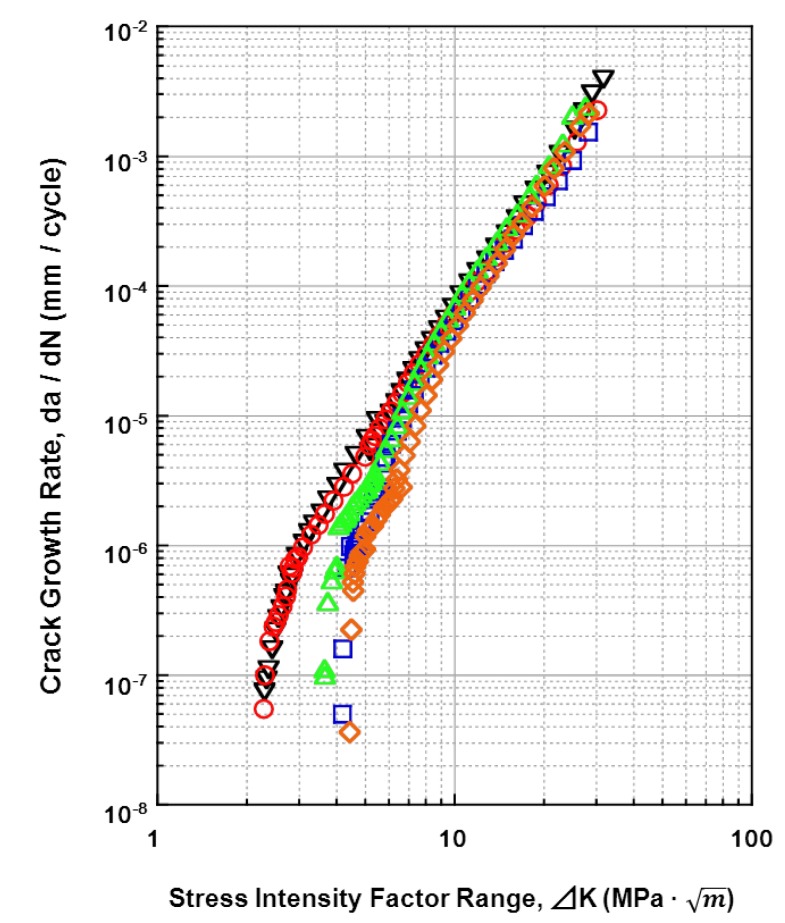
Effects of cutting direction on fatigue crack growth rate obtained by fatigue crack test of annealed and aged Ti-15Zr-4Nb-4Ta alloys in air.

**Figure 16:**
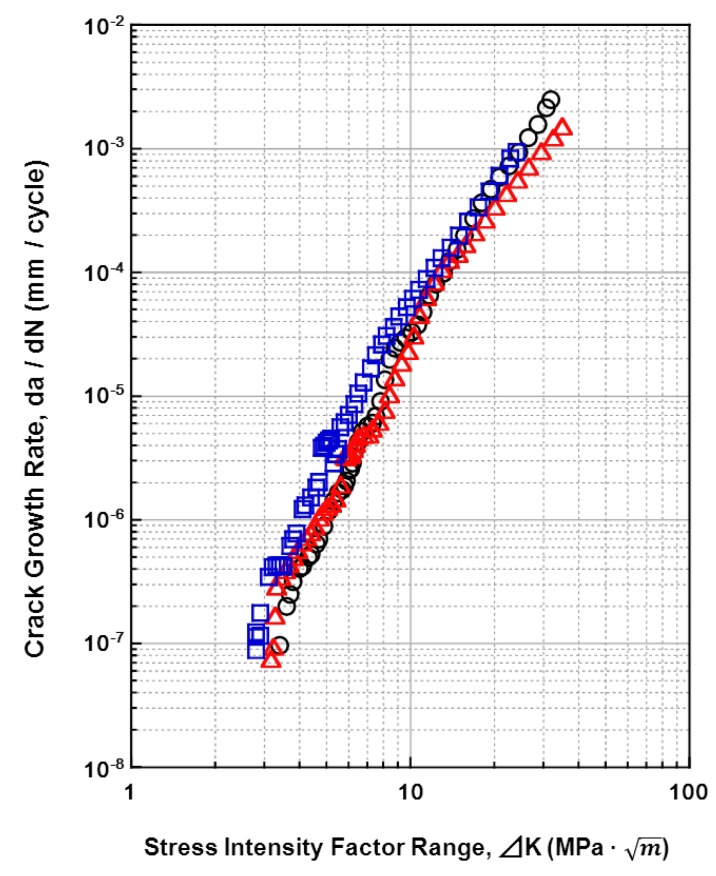
Effects of heat treatment on fatigue crack growth rate obtained by fatigue crack growth test of β-annealed and α-β-forged Ti-15Zr-4Nb-4Ta alloys in air.

**Figure 17:**
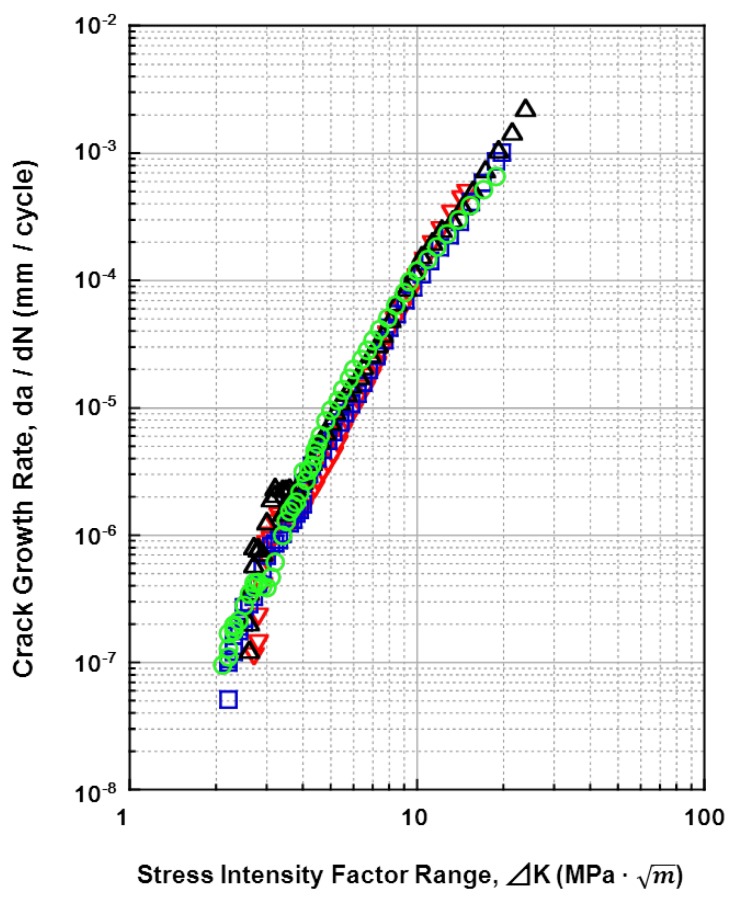
Effect of 0.9% NaCl on fatigue crack growth rate obtained by fatigue crack test of annealed Ti-15Zr-4Nb-4Ta alloy.

**Figure 18:**
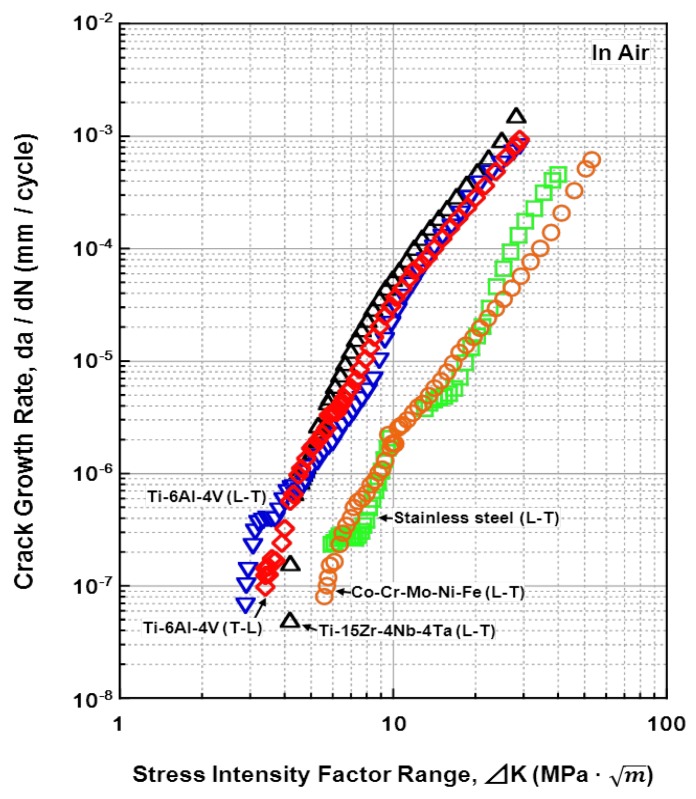
Effects of material on fatigue crack growth rates of specimens cut in L-T and T-L directions.

The relationships between the fatigue crack growth rates (da/dN) of all the materials tested and the stress-intensity factor range (ΔK) (fatigue crack growth rate) are shown in Figure 15, Figure 16, Figure 17 and Figure 18. Figure 15 shows the effects of the specimen cutting direction (S-L, S-T, or L-T) on the fatigue-crack growth rates of the specimens cut from the annealed and aged Ti-15Zr-4Nb-4Ta alloys. The threshold stress-intensity factor range ΔK_th_ of the aged alloy was slightly larger (excellent) than that of the annealed alloy. On the other hand, there was no difference in the fatigue crack growth rate between S-L, S-T, and L-T in the stable growth range or at ΔK_th_. Figure 16 shows the effects of forging and heat treatment on the fatigue-crack growth rates of the L-T and T-L specimens cut from the β-annealed and α-β-forged Ti-15Zr-4Nb-4Ta alloys. It is clear that the effects of forging and heat treatment were negligible. Figure 17 shows the effect of 0.9% NaCl on the fatigue-crack growth rates of the L-T and T-L specimens cut from the annealed Ti-15Zr-4Nb-4Ta alloys after α-β forging. Although the fatigue crack growth rate in 0.9% NaCl indicated was equal to that in air, the ΔK_th_ values for both L-T and T-L in 0.9%NaCl were slightly larger. This is probably because a clogged crack tip with a corrosion product induced oxide-induced crack closure. The L-T specimen was considered to have the most appropriate cutting direction from the results. A comparison between the fatigue-crack growth rates of the annealed Ti-15Zr-4Nb-4Ta alloy, annealed Ti-6Al-4V alloy, solution-treated stainless steel, and annealed low-C Co-Cr-Mo-Ni-Fe alloy is shown in Figure 18. The fatigue-crack growth rate of the Ti-15Zr-4Nb-4Ta alloy was similar to that of the Ti-6Al-4V alloy. On the other hand, the ΔK values of the stainless steel and Co-Cr-Mo-Ni-Fe alloy were much larger than those of the Ti alloys at 10^−3^ mm/cycle to 10^−7^ mm/cycle. The reason for this is that the Young’s moduli (E) of the stainless steel and Co-Cr-Mo-Ni-Fe alloy are higher than those of the Ti alloys. The ΔK_th_ of the Ti-6Al-4V alloy was smaller than the literature value (approximately 8 MPa $\sqrt{\text{m}}$) [54]. The reason for this may be the effect of the manufacturing process of the alloy. The material constants C and m of the implantable metals determined by the Paris equation (Region II) in the fatigue-crack growth test are summarized in Table 5. The value of m of the Ti-6Al-4V alloy in this study was close to that of Ti-6Al-4V-0.1Ru alloy reported in the literature (4 to 5) [55], whereas the C values were slightly different between these Ti alloys [55].

The maximum stress amplitude (Δσ_th_) at which no crack propagation occurs, estimated from ΔK_th_, was compared with the amplitude of fatigue strength (σ_n_ × 0.9) at 10^7^ cycles obtained from the V-notched rod specimen. The relationship between ΔK_th_ and Δσ_th_ is expressed as ΔK_th_* =* Δσ_th_ $\sqrt{\pi (d/2)}$ *f*, where *f* is the correction factor of the specimen and crack configuration. *f* could be determined using the Stress Analysis of Cracks Handbook [56]. The relationship between the maximum stress σ_th_ (Δσ_th_/0.9) used in this work and ΔK_th_ is expressed by σ_th_ = ΔK_th_/*f*(d/D)/$\sqrt{\pi (d/2)}$/0.9; here,

(7) $$f(\text{d}/\text{D})=1/2[1+1/2(\text{d}/\text{D})+3/8{\left(\text{d}/\text{D}\right)}^{2}-0.363{\left(\text{d}/\text{D}\right)}^{3}+0.731{\left(\text{d}/\text{D}\right)}^{4}]/\sqrt{1\;-\text{ d}/\text{D}}$$

where d/D = 0.8, d is the notch root diameter, and D is the specimen diameter. The *f* (d/D) of the V-notched rod specimen used in this work (Figure 2c) is 0.39 [57]. The value of σ_th_ calculated using ΔK_th_ was fairly small compared with the fatigue strength (σ_n_) at 10^7^ cycles measured by the notch fatigue test. For example, the value of σ_th_ of the annealed Ti-15Zr-4Nb-4Ta alloy calculated with ΔK_th_ = 2.2 MPa $\sqrt{m}$ was approximately 64 MPa, which was approximately one-fifth of the amplitude of the notch fatigue strength at 10^7^ cycles (Δσ_n_ = σ_n_ × 0.9 = 360 MPa), as shown in Figure 14. The reason for this might be related to the increase in notch tensile strength, as shown in Table 2. In addition, it might be related to the differences in notch configuration and machining conditions between the notched and CT specimens at the crack tip. The slope (m’) of the S-N curve of log σ_max_ *vs.* log N_f_ plotted in the low-cycle region (approximately < 5 × 10^4^ cycles) estimated from the S-N curves obtained by the V-notched rod fatigue test is shown in Table 5 in comparison with the constant m. It is considered that these values were close to the value of m determined using the Paris equation (Region II) in the fatigue-crack growth test [37]. However, m’ is considerably smaller than the constant m determined by the Paris equation. From the results obtained in this work, it is suggested that the notched fatigue test with a stress concentration factor at concentrated parts of implant products, such as metallic bone plates, nails, screws, spinal implants, and artificial hip stem products, is more important than the fatigue crack growth test for durability evaluation. Thus, it is considered that the effectiveness of the notched fatigue strength is greater than that of the fatigue crack growth rate for the durability evaluation of implant products.

**Table 5:** Comparison of m and c obtained by fatigue crack growth test (L-T) of various metals.

Alloy	c	m	m'
Ti-15Zr-4Nb-4Ta			
Annealed (L-T)	3.44 × 10^−8^	3.48	1.3
0.9%NaCl (L-T)	1.67 × 10^−8^	4.48	
Aged (L-T)	0.83 × 10^−8^	3.73	0.6
Ti-6Al-4V			
Annealed	3.07 × 10^−8^	3.0	1.4
Stainless (ISO 5832)			
Solution-treated	1.94 × 10^−12^	5.32	0.9
Co-Cr-Mo-Ni-Fe			
Annealed	0.15 × 10^−8^	3.24	0.83

## 4. Conclusions

The mechanical properties, fatigue strengths, and fatigue crack growth rates of Ti-15Zr-4Nb-4Ta alloy were compared with those of other implantable metals, namely, commercially pure (C.P.) grade 4 Ti, Ti-6Al-4V, Co-Cr-Mo, Co-Cr-Mo-Ni-Fe, and Co-Cr-W-Ni alloys, and stainless steels. The highly biocompatible Ti-15Zr-4Nb-4Ta alloy exhibited an excellent balance between strength and ductility. Mechanical strengths were also markedly increased by a combination of manufacturing processes and heat treatment. In particular, the strength of the 20% cold-worked C.P. grade 4 Ti was close to that of the Ti alloys. The mechanical strengths of the 20% cold-worked stainless steel, and hot-forged and warm-worked Co-Cr-Mo, cold-drawn Co-Cr-Mo-Ni-Fe, and aged Ti-15Zr-4Nb-4Ta alloys were markedly higher than those of the solution-treated and annealed materials. On the other hand, the T.E. and R.A. values of these materials decreased with an increase in mechanical strength. The notched tensile strength (σ_NTS_) of the Ti-15Zr-4Nb-4Ta alloy was also much higher than those of the smooth specimens. The tension-to-tension fatigue strength of the annealed Ti-15Zr-4Nb-4Ta alloy rod at 10^7^ cycles was approximately 740 MPa, which was slightly higher than that (680 MPa) of the Ti-6Al-4V alloy. The fatigue strength of this alloy was much improved by aging treatment after solution treatment. The fatigue strength of the stainless steel at 10^7 ^cycles was markedly improved by 20% cold working and became close to that of the Ti alloy. The fatigue strength of the Co-Cr-Mo alloy at 10^7 ^cycles was markedly increased by warm working or hot forging and was considerably higher than that of the Ti alloy. The fatigue strengths of the high-C and low-C Co-Cr-Mo-Ni-Fe alloys were markedly improved by cold-drawing compared with that of the annealed alloy; the strength of these alloys was approximately 1100 MPa. The ratios of fatigue the strength (σ_FS_) at 10^7 ^cycles to the ultimate tensile strength (σ_FS_/σ_UTS_) of all the materials were within the 0.5 to 0.9 range. The annealed and aged Ti-15Zr-4Nb-4Ta alloy rods exhibited high σ_FS_/σ_UTS_ values of approximately 0.8 and 0.9, respectively. The effect of the V-notch on the S-N curves of the solution-treated stainless steel was small. However, the fatigue strength of the 20% cold-worked stainless steel markedly decreased with the existence of the V-notch. The notch fatigue strengths of the 20% cold-worked C.P. grade 4 Ti, and annealed and aged Ti-15Zr-4Nb-4Ta and annealed Ti-6Al-4V alloys at 10^7 ^cycles were considerably less than those of the smooth specimens. The fatigue crack growth rate in 0.9% NaCl was equal to that determined in air. The threshold stress-intensity factor range (ΔK_th_) of the aged alloy was slightly larger than that of the annealed alloy. There was no difference in the fatigue crack growth rate between the S-L, S-T, T-L, and L-T directions in the stable growth range or at ΔK_th_. The L-T specimen was considered to have the most appropriate cutting direction from the results. The reason for this is that the stainless steel and Co-Cr-Mo-Ni-Fe alloy had larger ΔK values than Ti alloys. The strength (σ_th_), which indicates no crack propagation, estimated using ΔK_th_ values, was fairly small compared with the fatigue strength at 10^7^ cycles (σ_n_) obtained by the notched fatigue test.

## Supplementary Materials

Supplementary File 1
