# Supplementary material for: Comparison of Fatigue Properties and Fatigue Crack Growth Rates of Various Implantable Metals
Source: Materials (Basel). 2012 Dec 19;5(12):2981–3005. doi: 10.3390/ma5122981 (PMC5449063; doi:10.3390/ma5122981)
Supplement: Supplementary File 1 [file materials-05-02981-s001.pdf]

Supplementary File

OPEN ACCESS

materials

ISSN 1996-1944

www.mdpi.com/journal/materials

Comparison of Fatigue Properties and Fatigue Crack Growth Rates of Various Implantable Metals

Yoshimitsu Okazaki

Advanced Biomaterials Group, Human Technology Research Institute, National Institute of Advanced Industrial Science and Technology, 1-1-1 Higashi, Tsukuba-shi, Ibaraki 305-8566, Japan

---

Due to an oversight by the author, in the research article [1] the legends of Figures 15, 16 and 17 on pages 2998 and 2999 indicating the type of metals used for the experiments were missing. Below are the complete figures.

Figure 15. Effects of cutting direction on fatigue crack growth rate obtained by fatigue crack test of annealed and aged Ti-15Zr-4Nb-4Ta alloys in air.

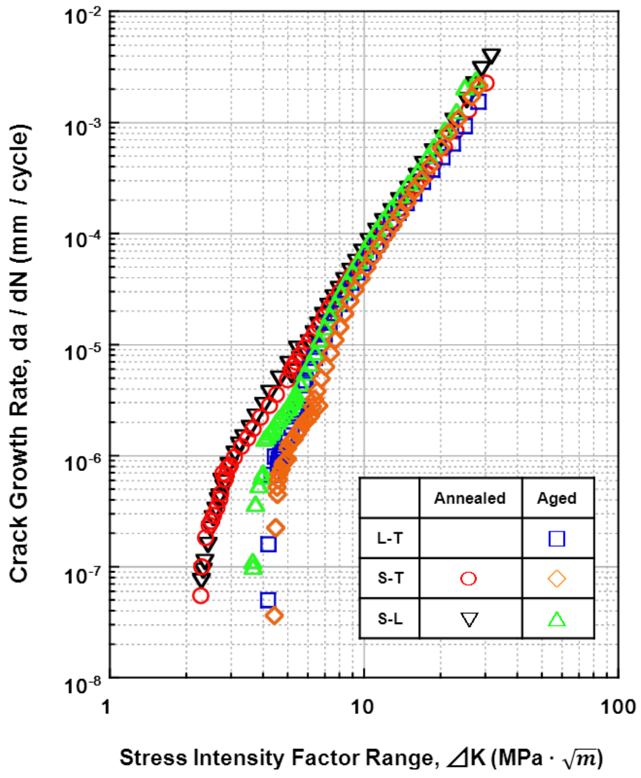

Figure 16. Effects of heat treatment on fatigue crack growth rate obtained by fatigue crack growth test of β-annealed and α-β-forged Ti-15Zr-4Nb-4Ta alloys in air.

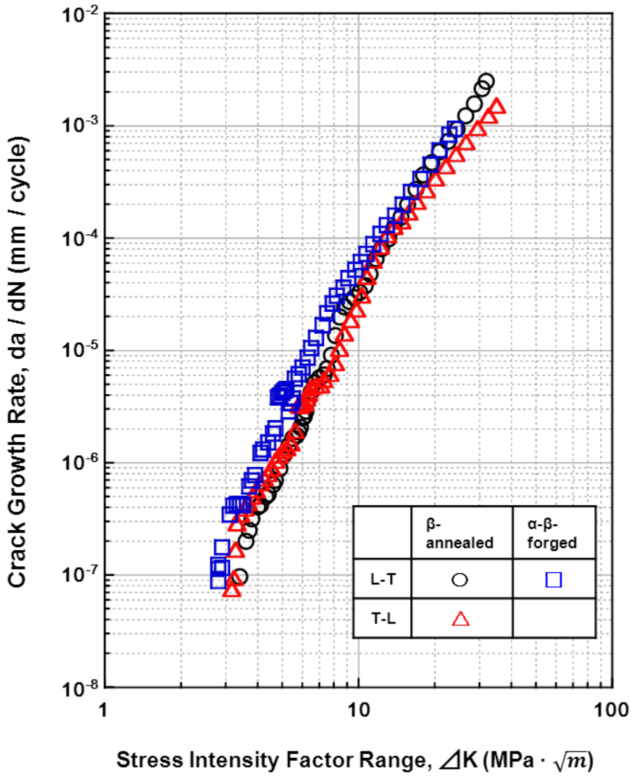

Figure 17. Effect of 0.9% NaCl on fatigue crack growth rate obtained by fatigue crack test of annealed Ti-15Zr-4Nb-4Ta alloy.

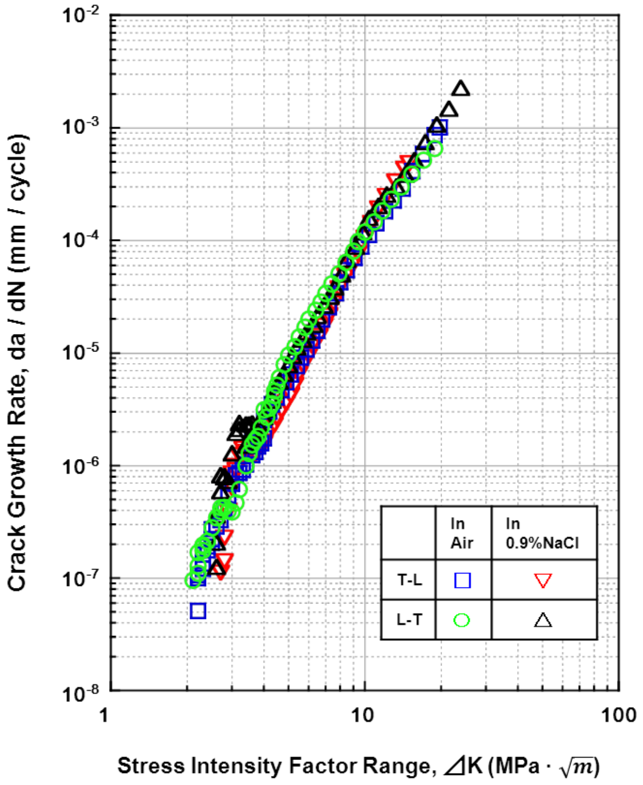

We apologize for any inconvenience this may have caused.

References

1. Okazaki, Y. Comparison of Fatigue Properties and Fatigue Crack Growth Rates of Various Implantable Metals. *Materials* **2012**, *5*, 2981-3005.

© 2013 by the authors; licensee MDPI, Basel, Switzerland. This article is an open access article distributed under the terms and conditions of the Creative Commons Attribution license (http://creativecommons.org/licenses/by/3.0/).
